# A Novel Approach for Analysis of Rocking Curve X-Ray Diffraction Imaging Data (RC-XRDI) on 4H-SiC Using Cumulative Integrated Intensity (CII) Method

**DOI:** 10.1007/s11664-025-11963-y

**Published:** 2025-05-09

**Authors:** Arash Estiri, Richard Bytheway, Tamzin Amanda Lafford, Oliver James Louis Fox, Andrew Graham, Claire Hurley, Vishal Ajit Shah

**Affiliations:** 1 School of Engineering, The University of Warwick, Coventry, CV4 7AL UK; 2 Bruker Ltd., Belmont Business Park, Durham, DH1 1TW UK; 3 B16 Test Beamline, Diamond Light Source, Didcot, OX11 0DE UK; 4 Physics Department, The University of Warwick, Coventry, CV4 7AL UK

**Keywords:** X-ray topography (XRT), x-ray diffraction imaging (XRDI), defects, silicon carbide, 4H-SiC, Gaussian

## Abstract

This paper presents a novel method of using cumulative integrated intensity (CII) to analyse rocking curve x-ray diffraction imaging (RC-XRDI) data. This method overcomes several limitations of traditional complex non-ideal curve fitting, which often results in inaccurate peak detection and full width at half maximum (FWHM) extraction. These complex non-ideal rocking curves arise in cases where additional features are present, such as peak splitting and multiple peaks. The application of the method also avoids the need for curve fitting and time-consuming calculations, allowing the extraction of peak widths at various normalized height-intensities (FWxM) and revealing extra information about defects. By analysing the broadening and peak position of the rocking curves for different defects, RC-XRDI provides insights into the nature and distribution of these defects within the material. Applied to RC-XRDI of a 4H-SiC 10 μm-thick homo-epitaxial layer on a substrate, the CII method was used to detect shifts in peak position and generate maps of full width at 1%, 10%, and 50% of maximum intensity, offering a detailed view of defect-induced broadening. Our results demonstrate that the CII method provides improved accuracy and requires fewer computations compared to curve-fitting techniques, making it particularly useful where precise defect characterization is critical. Moreover, background intensity was detected pixel-by-pixel using cubic smoothing splines, and the CII method provided robust validation for the precision of this background detection.

## Introduction

Rocking curve x-ray diffraction imaging (RC-XRDI) offers detailed insights into strain, mosaicity and defects within a crystal lattice by rotating the sample around its Bragg angle and capturing high-resolution two-dimensional images.^1–4^ This technique enables researchers to evaluate material quality by mapping local rocking curves in each pixel of the image across an area of a sample. ^1,3^ This capability is critical for semiconductor materials which are commercialized and are maturing, but still have relatively high defect densities, such as silicon carbide (SiC). ^5,6^

4H-SiC is one of the common polytypes of SiC that is favoured for power electronic devices due to its high critical electric field (2.8 MV cm^−1^), wide bandgap (3.2 eV) and high thermal conductivity (300 W K^−1^ cm^−1^), and has gained popularity for the development of medium- to high-voltage devices (>1.2 kV) with commercial off-the-shelf devices routinely available on the market. The largest demand for these devices is in manufacture of hybrid and fully electric cars, with the prediction that the SiC power device market will be worth $10 billion by 2030. ^7,8^ However, the high density of defects, like threading screw dislocations (TSDs, 10^2^−10^3^ cm^−2^), threading edge dislocations (TEDs, 10^3^−10^4^ cm^−2^),^9^ stacking faults (SFs, < 10 cm^−2^),^10^ and basal plane dislocations (BPDs, 10^1^−10^3^ cm^−2^) ^9,11^ can significantly degrade device performance, yield and reliability. ^5,12,13^ Therefore, identification of additional information about the defects will aid in developing strategies for their reduction and mitigation. ^14^

The full width at half maximum (FWHM) of the rocking curve has been employed to assess the quality of crystalline materials,^1,3,4,13^ with the theoretical FWHM of the reflectivity curve of a perfect crystal serving as a benchmark. ^15^ RC-XRDI analysis has often relied heavily on curve fitting, which is suitable only for idealized, symmetric curves.^16,17^ Due to the low beam divergence, rocking curves obtained from synchrotron beam diffraction often deviate from a Gaussian profile, particularly for samples with regions containing complex defect structures, which produces multiple peaks, peak splitting, or asymmetric broadening in RC-XRDI. In such cases, curve-fitting methods require suitable fitting models and become inaccurate, demanding computationally intensive processing and risking erroneous interpretations of data. The quantitative effect of defects can be assessed by calculating the peak position^1,16–18^ and peak broadening using FWHM to measure approximately the dislocation density .^19^

To address these shortcomings, we propose a cumulative integrated intensity (CII) method to analyse RC-XRDI data. This approach eliminates the need for calculation of coefficient of determination (R-squared), enabling direct measurement of full width at a fraction of maximum intensity (FWxM). The CII method captures essential information on defect-induced broadening in RC-XRDI by revealing how each defect type contributes differently to peak broadening, which is often obscured or ignored in traditional analysis.

## Experimental Configuration and Data Collection

4H-SiC homoepitaxial layers were grown at Warwick on 100 mm-diameter, 4° off-axis, 325 µm-thick SiC wafers using an LPE AciS M8 chemical vapour deposition (CVD) reactor. The homoepitaxial layers of nominal 10 µm thickness and nitrogen doping of 3 $\times$ 10^14^ cm^−3^ were grown using trichlorosilane (HCl_3_Si, TCS) and ethylene (C_2_H_4_) with growth rates of 30 µm hr^−1^.^20,21^

**Fig. 1:**
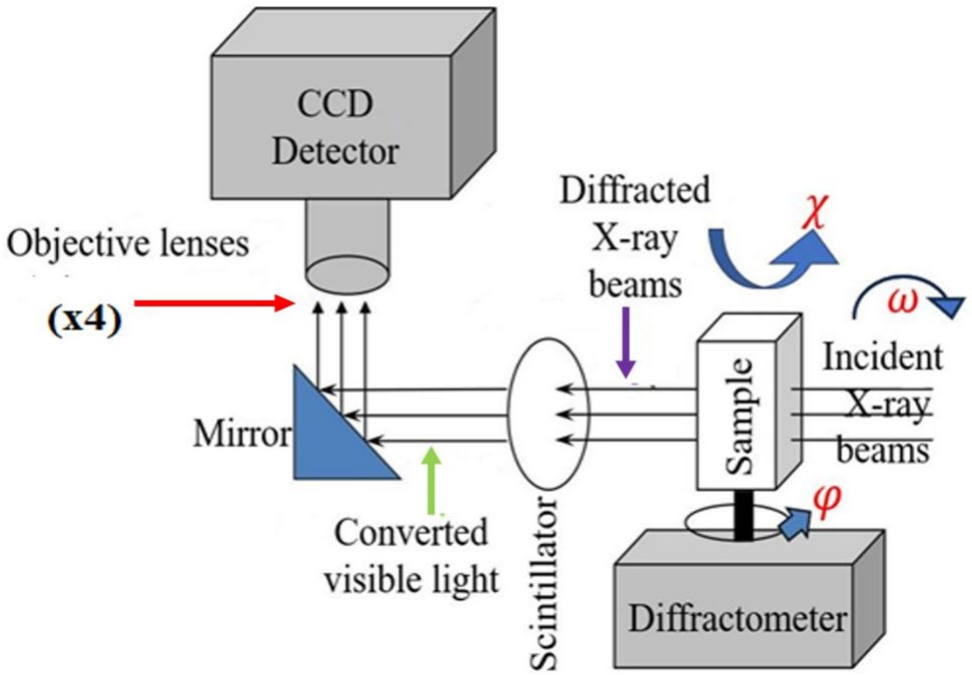
Experimental configuration for rocking curve x-ray diffraction imaging (RC-XRDI) at the B16 Test Beamline at DLS. Adapted from Ref. 22. Adapted from 22 under the terms of the Creative Commons Attribution 4.0 International License http://creativecommons.org/licenses/by/4.0/

RC-XRDI was performed on $\left(1 1 \overline{2} 0\right)$ crystal planes of the 4H-SiC sample using a synchrotron x-ray source at the B16 Test Beamline at the Diamond Light Source (DLS), configured to produce monochromatic radiation with a wavelength of 0.709 Å, corresponding to the Mo-Kα_1_ characteristic radiation. In addition, synchrotron white beam x-ray diffraction imaging (SWB-XRDI), which uses the full spectrum of the bending magnet source, was utilized to acquire high-resolution images of the samples for different crystallographic planes. The configuration of the 4H-SiC sample with respect to the beam is shown in Fig. 1, where phi (*φ*) is the rotation around an axis normal to the sample, while chi (χ) is the rotation around the beam axis. A two-dimensional imaging detector consisted of a 35 µm-thick Ce:YAG scintillator coupled with a PCO-4000 CCD camera using a ×4 objective lens, which resulted in an effective pixel size of 2.25 µm and a pixel count of 2244 $\times$ 2660 per image. The sample was configured around a eucentric point in transmission geometry, where omega (*ω*) was rocked around the diffraction peak position a range of *ω* = 0.05° with a step size of 0.0005° to collect diffraction data. As the sample was rocked, a high-resolution image of the diffracted beam was collected at each omega position, where each pixel in the detector corresponds to a unique local diffraction area.

## Data Analysis

### Cumulative Integrated Intensity (CII) Method

**Fig. 2:**
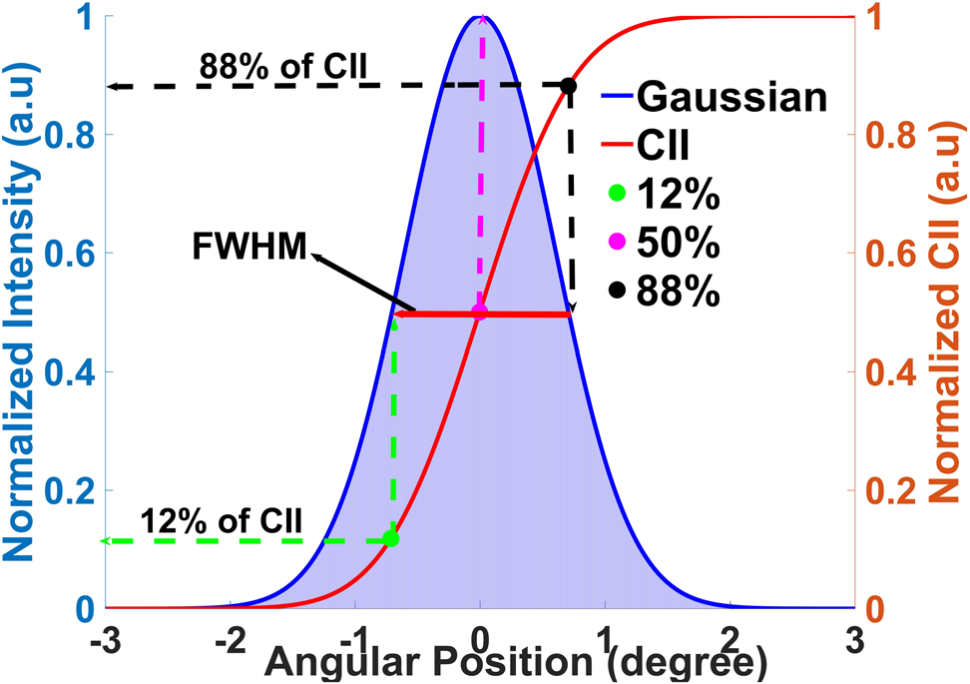
Correlation between the CII and an ideal Gaussian curve. 50% of CII represents the peak position of a Gaussian curve, while 12% and 88% are the lower and upper bounds of the FWHM.

Conventional curve-fitting methods like Gaussian and Lorentzian are inadequate for non-Gaussian rocking curve analysis, especially in regions with multiple peaks, splitting, or asymmetry caused by defects. To overcome these limitations, we have developed a CII method for direct measurement of FWxM, treating all the features as belonging to one diffraction peak. The process begins by first determining a threshold intensity as the background signal in each pixel by (1) analysing a single rocking curve image and leaving the background data in each pixel, then (2) using cubic smoothing spline fitting to find the fit for the background data, ^23^ and finally, (3) subtracting this fit from the rocking curve data. This final step is crucial for minimizing unwanted intensities and ensuring the integrity of the diffraction signal. Then the CII is calculated by multiplying each adjusted intensity value by the width of the angular step size and integrating these products across the angular range. This method captures the contribution of each angle to the total intensity. Figure 2 illustrates the correlation between a Gaussian curve (blue) and its cumulative integrated intensity (CII, red curve). Using equation (1) the FWxM for Gaussian curve is calculated, where x is the fractional intensity of the maximum, and σ is the standard deviation. Using the half-normalized (HN) width equation (2), the lower and upper cumulative distribution function (CDF) bounds are derived via the error function (erf), with the formulas (3) and (4), respectively. For instance, 12% and 88% of CII correspond to the lower and upper bounds of the FWHM, while the peak position of the Gaussian curve is consistently captured at 50% of the CII. The relationships between some of the corresponding points on the CII and the Gaussian curve are detailed in Table I.

1 $$ {\text{FW}} \times {\text{M }} = { 2}\sigma \sqrt {( - 2lnx)}, $$

2 $$ {\text{Half}} - {\text{Normalized }}\left( {{\text{HS}}} \right){\text{ FWxM }} = {\text{ HN}}{\text{FWxM }} = {\text{FWxM }}/{ 2}\sigma \, = \sqrt { - 2lnx}. $$

**Table I:** The relationship between the CII and Gaussian curve

FWxM Gaussian	LB CII Value (%)	UP CII Value (%)
0.01	0.12	99.8
0.1	1.6	98.4
0.2	3.6	96.3
0.3	6.0	93.9
0.5 (H)	11.9	88.0
0.7	19.9	80.0

3 $$ {\text{Lower Bound }}({\text{LB}}){\text{ of CII }} = 0.5 \times \left( {1 + {\text{erf }}\left( {\frac{{ - {\text{HN}}{\text{FW}} \times {\text{M}}}}{{\sqrt 2 }}} \right)} \right), $$

4 $$ {\text{Upper Bound }}({\text{UB}}){\text{ of CII }} = 0.5 \times \left( {1 + {\text{erf }}\left( {\frac{{ - {\text{HN}}{\text{FW}} \times {\text{M}}}}{{\sqrt 2 }}} \right)} \right), $$

### Influence of Background Detection on CII Shape

**Fig. 3:**
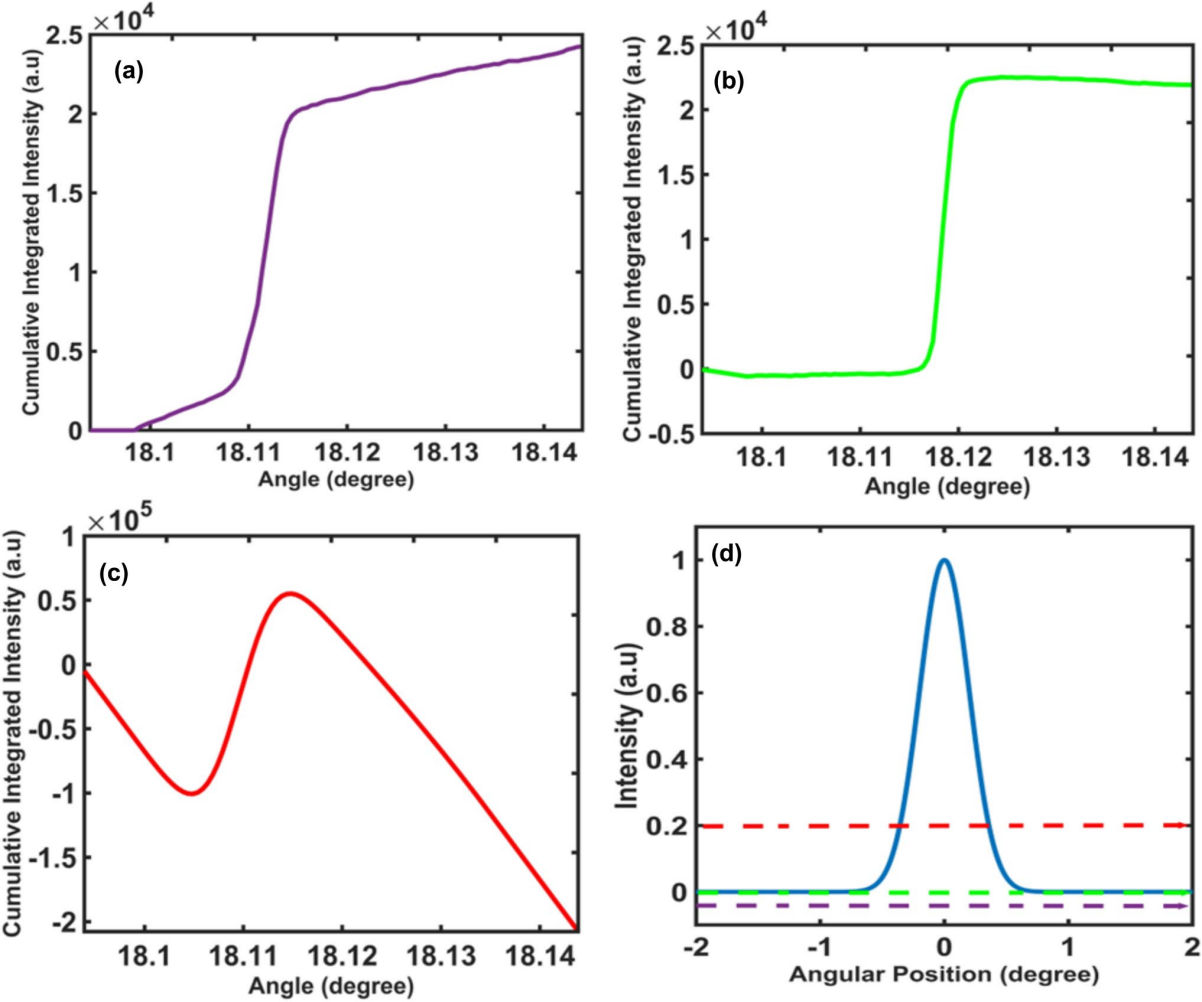
Correlation between background detection and the CII shape (a) underestimated, (b) correctly estimated, (c) overestimated, and (d) a simulated ideal rocking curve to show the position of underestimated (purple dashed line), correctly estimated (green dashed line), and overestimated (red dashed line) background detection.

Accurate background detection is crucial for ensuring that the CII accurately reflects the diffraction peaks. The cubic smoothing spline technique was used to fit a piecewise cubic polynomial to the rocking curve data to capture the general trend of background, then the detected background signal was identified outside the peak and subtracted from the data. As the background behaviour is unknown with respect to whether it follows a linear or nonlinear pattern, a cubic smoothing spline technique was applied to ensure accurate background removal on a pixel-by-pixel basis while maintaining the overall shape of the rocking curve. Properly identifying and removing background intensity is critical to preserving the CDF shape of the CII method, as improper estimation leads to inaccurate conclusions of the measured peak widths and peak positions. Underestimating the background, as shown in Fig. 3a, results in a positive slope in the CII tails, while as depicted in Fig. 3b, accurate background subtraction produces tails with a near-zero slope or smooth plateau behaviour in the tails, which is crucial for reliable material characterization and quantitative analysis. In contrast, Fig. 3c demonstrates that overestimating the background causes the tails to exhibit a negative slope. The positions of underestimated, correctly estimated, and overestimated background detection in a simulated ideal rocking curve data are presented in Fig. 3d with purple, green and red colour dashed lines, respectively.

## Results and Discussion

### CII Comparison with Gaussian Fitting

A direct comparison between the CII and the traditional Gaussian curve-fitting technique demonstrates the limitations of the latter, especially in handling non-Gaussian curves. Traditional curve fitting relies on idealized assumptions about peak shapes, which can result in inaccuracies when analysing rocking curve data that do not meet these assumptions. In contrast, the flexibility of this novel method in accommodating complex structures (multiple and split peaks) makes it a superior alternative for defect analysis. This versatility requires less computation and decreases processing time, on the scale of 10^3^ times faster than a Gaussian approach. However, with modern computation hardware this may not be a concern unless data sets are very large, as is the case for the more than 6 M pixel count camera used in this study. It also significantly reduces the risk of errors in data analysis, such as inaccuracies in detection of peak position.

**Fig. 4:**
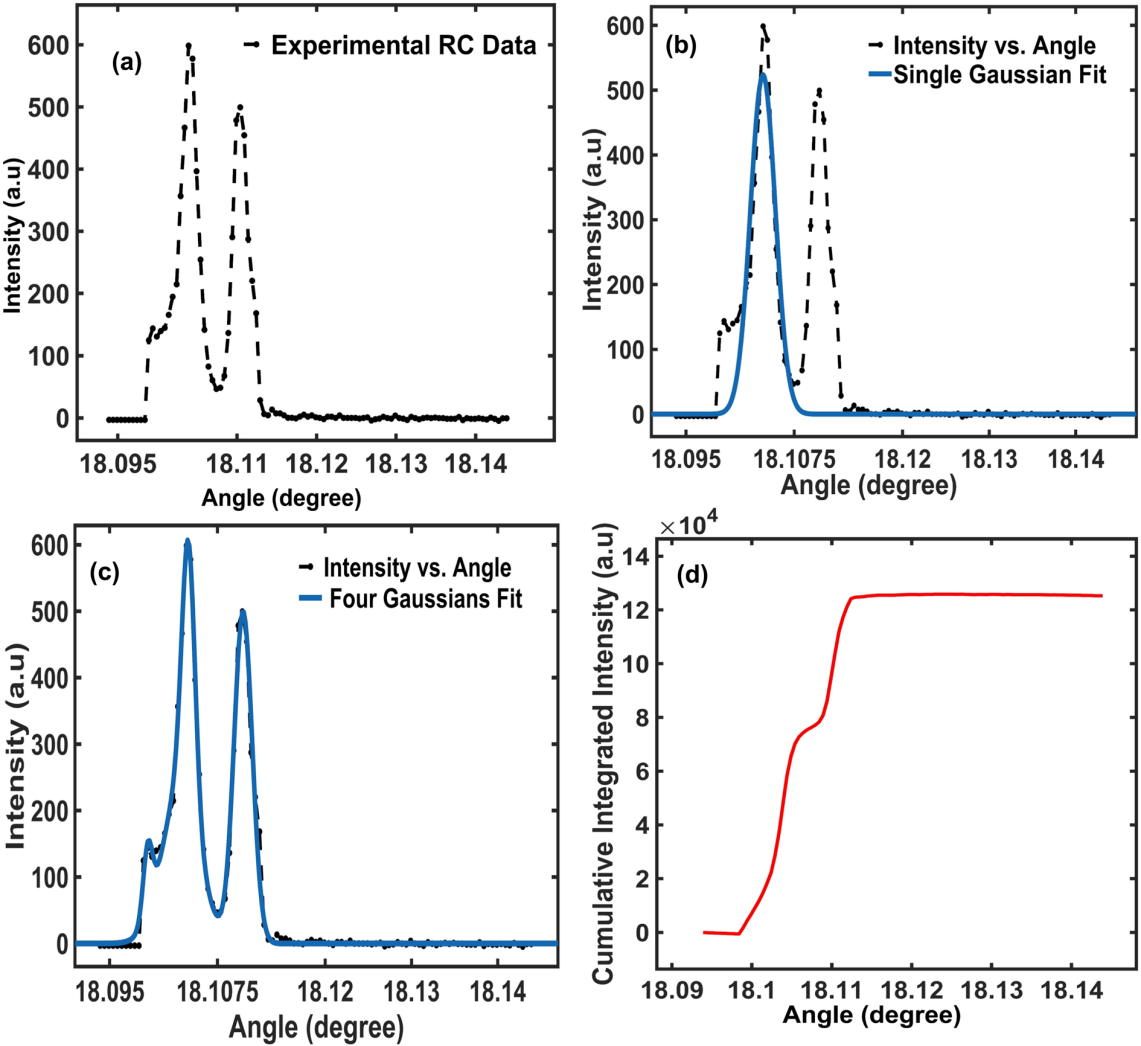
Comparison between the effectiveness of Gaussian curve fitting versus the CII method. (a) Experimental rocking curve data, fitting (b) a single Gaussian curve fitting to (a), (c) four Gaussians to (a), (d) the CII of (a).

In Fig. 4a, the experimental RC-XRDI are presented by dashed lines from a region containing a 3C-SiC polytype defect. In Fig. 4b, the same data from Fig. 4a are presented along with a single Gaussian fit which oversimplifies the data and does not adequately reflect the variation in defect-induced broadening; the FWHM of the single Gaussian does not present the variation in the rocking curve data. In contrast, Fig. 4c illustrates the result of fitting four Gaussians to the same data, to better capture the complexity of the rocking curve, but a FWHM from the four Gaussians model does not accurately reflect the FWHM in Fig. 4a. However, even the four Gaussians model, whose coefficient of determination is close to 1, fails to capture the subtle nuances in the data, particularly in regions of the rocking curve where non-Gaussian characteristics dominate. This highlights the inherent challenge of Gaussian fitting approaches: it is difficult to determine whether a single-Gaussian or multi-Gaussian approach is more appropriate for representing the rocking curve data accurately. Illustration of the efficacy of the CII method is presented in Fig. 4d, which directly handles multiple peaks within the data without relying on complex fitting algorithms. The significant advantage of this method is its ability to simplify complex peak structures, e.g., where two well-separated peaks (Fig. 4a) are represented as distinct steps and treated as a single peak (Fig. 4d). This method is most effective when consolidating multiple peaks or noisy data into a single peak, prioritizing overall broadening and defect-induced feature distribution over resolving individual peaks.

In our study, the observed multiple peaks arise from the detector-to-sample distance rather than material heterogeneity.^12^ The CII approach allows noisy data to be treated as a single peak, ensuring more reliable analysis without the need for individual peak separation. Although this limitation can be mitigated through programming that defines specific ranges to treat each peak separately. This makes the CII approach a more robust and efficient solution, particularly for complex datasets where Gaussian fitting can be prone to inaccuracies. Furthermore, unlike breadth, which provides only a single integrated measure and lacks flexibility in capturing peak broadening at different intensity levels, the CII method allows for the extraction of peak width at any fraction of maximum intensity. It is independent of peak shape, eliminates fitting errors, and enables a more comprehensive approach to defect characterization. This adaptability makes CII a more precise and versatile alternative, ensuring reliable peak width analysis across varying intensity thresholds. FWxM values can be readily read off using the intensity proportions given in Table I.

Table II compares the advantages and limitations of the CII and curve-fitting methods.

**Table II:** Comparison of the advantages and the limitations of the CII and curve-fitting methods

Feature	CII method	Curve-fitting method (e.g. Gaussian)
Purpose	Designed for a reliable and rapid statistical mapping of defect distributions and FWxM in rocking curves, emphasizing broadening effects	Primarily used for individual peak resolution, focusing on detailed characterization of peak parameters like position and FWHM
Handling of peak shapes	Handles any peak shape, e.g., non-Gaussian and complex peak shapes, including asymmetric or split peaks	Requires peaks to be modelled using idealized shapes (e.g. Gaussian, Lorentzian). Struggles with highly asymmetric or split peaks
Complexity	Simple to implement without requiring iterative algorithms or curve model selection	Requires selecting a fitting model and optimizing parameters, which can lead to errors if the model is inappropriate
Limitations	Not suitable for separating multiple peaks, as it treats them as one and requires a predefined integration range	Struggles with non-Gaussian or complex peak shapes. It can be prone to overfitting or misfitting
Best use cases	Mapping defect distributions, analysing long-range effects of defects, and identifying defect-induced broadening	Detailed peak analysis for individual peaks in high-resolution datasets or where peak resolution is critical if it is well-modelled in advanced

### Peak Position Mapping

**Fig. 5:**
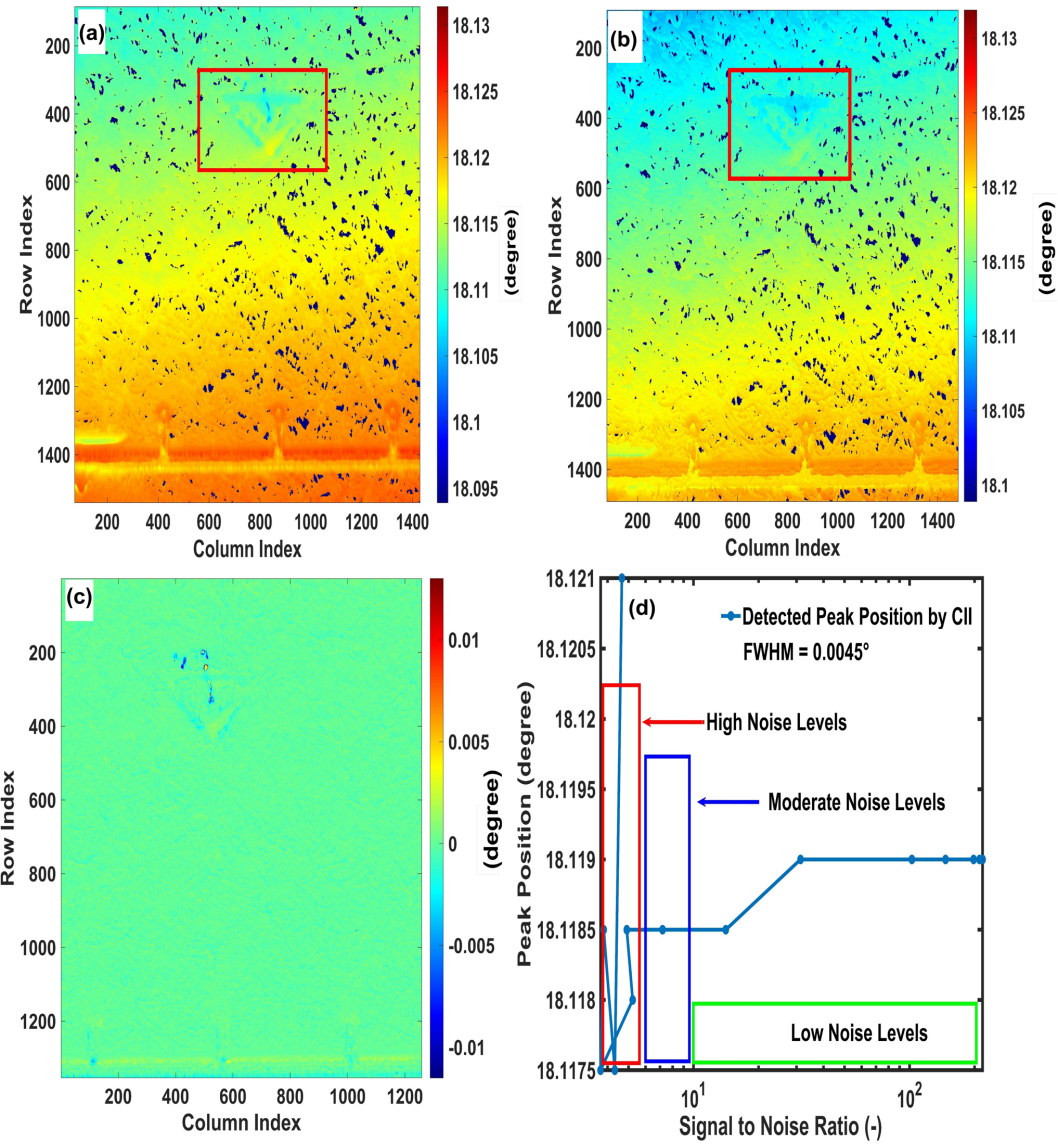
Comparison between peak position maps obtained by (a) the CII, and (b) MATLAB's *findpeaks* function; the red rectangles indicate a region with 3C-SiC polytype. (c) Colormap showing detection of peak position difference by CII method and MATLAB’s *findpeaks* function. (d) Impact of applying different signal-to-noise ratio (SNR) on accuracy of peak position detection by CII.

Peak position detection from the rocking curve is essential for assessing the local rotation of the crystal lattice with respect to the beam direction.^3,4,24^ Shifts in peak position reveal important information about the tilt and twist of the crystal lattice, rather than direct strain.^3,24^ In our analysis, a region of 3C-polytype was identified and highlighted with red rectangles in Fig. 5a and b. This defect acts as a non-killer defect that lowers breakdown voltage in Schottky barrier diodes (SBD) and increases forward voltage in p–n diodes.^6^ Therefore, it impacts device performance, which is important in power applications requiring reliable high-voltage performance.^5,6^ This defect identified in our study originated during the epitaxial growth of 4H-SiC which is potentially caused by non-ideal growth conditions, such as variations in temperature, precursor flow rates ratios, or other process-related factors.^25^

Peak position detection by the CII (Fig. 5a) method was validated against MATLAB’s *findpeaks* function (Fig. 5b) ^26^ by which is determined the maximum intensity and its corresponding angle (peak position) in each pixel. Both approaches yield similar results as confirmed by the green regions in Fig. 5c.

A comprehensive pixel by pixel analysis over the entire region demonstrates that the CII method achieves a mean absolute difference (MAD) of 0.0227% in detection of peak position. This discrepancy by CII indicates a peak position detection difference corresponding to a range of ± 3 $\times$ step size (0.0005°), highlighting that the CII method still effectively identifies peak position. This can be attributed to the presence of noise within certain pixels (Fig. 5a) and we propose CII as a way of dealing with such a case. A short distance between the detector and the sample in RC-XRDI improves spatial angular resolution, directing the diffracted beam to its intended pixel rather than neighbouring ones. This improves angular measurements by eliminating the occurrence of noise. ^12^ However, dealing with noise is another good reason to use CII.

Existence of noise and split peaks highlights that traditional peak detection methods identify only the main peak, disregarding smaller peaks and peak shoulders. This supports our earlier claim that conventional methods, such as Gaussian fitting, are insufficient for analysing complex data. By contrast, the CII method effectively treats these additional details, providing more robust and comprehensive peak position detection. To explore the influence of noise on the CII method’s precision, a simulated graph with a known FWHM (0.0045°), as presented in Fig. 5d, illustrates the effect of varying the signal-to-noise ratio (SNR) on the accuracy of the CII method in detecting a known peak position (18.119°). CII accurately detects the peak position when SNR is higher than 10^1^, corresponding to low noise levels, but shows pronounced deviations at high noise levels. Regions within the sample (Fig. 5a and b) where Bragg's law is not satisfied are indicated by dark-blue dots, identified by setting a threshold intensity corresponding to 2% of the maximum intensity. These regions signify areas where diffraction is unsuitable for CII analysis, possibly indicating significant structural deviations that require further investigation.

### FW×M Mapping

**Fig. 6:**
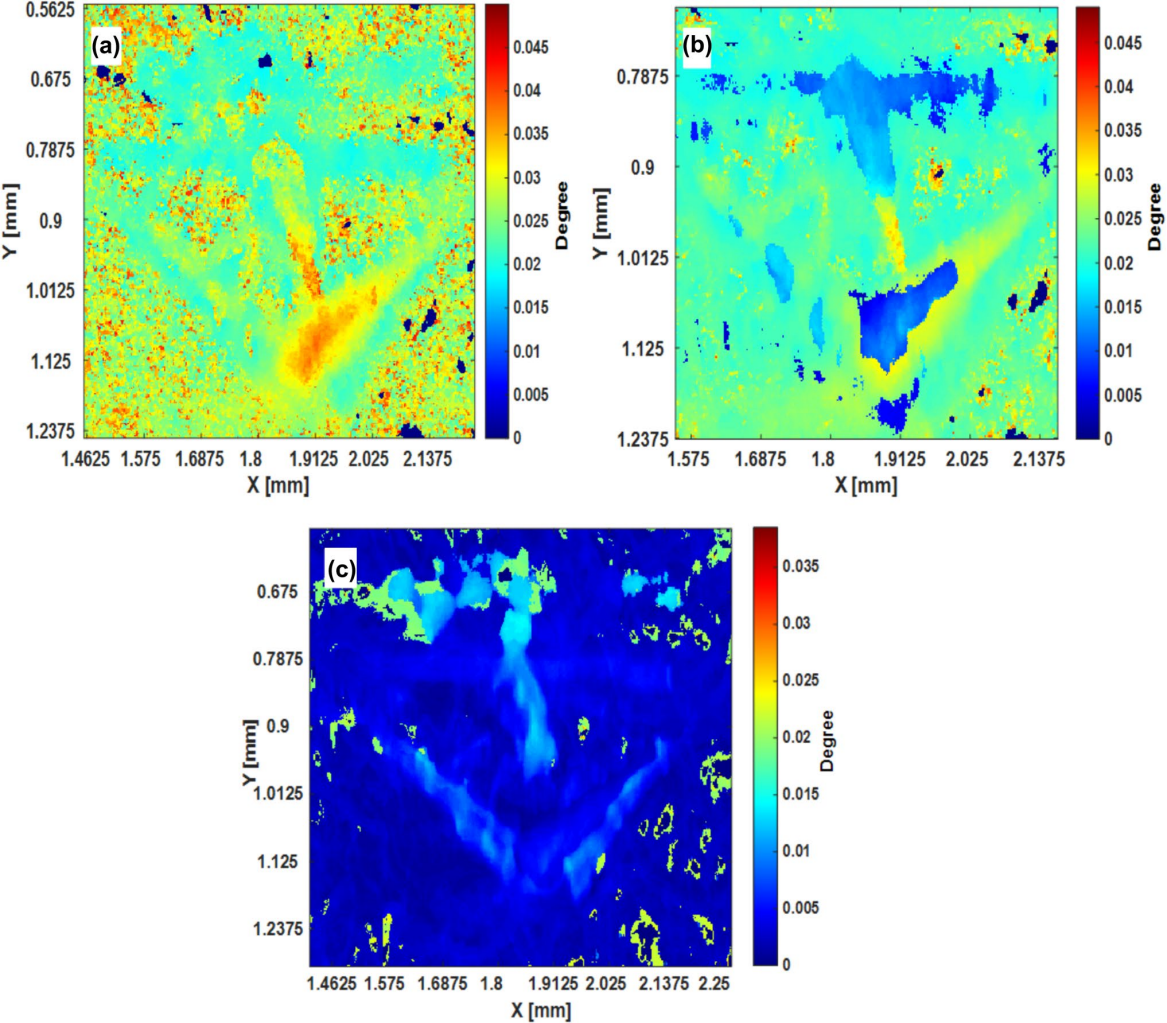
Full width at different fractions of maximum intensity (FWxM): (a) 1%, (b) 10%, and (c) 50%.

The influence of the 3C-SiC polytype defect on full width at 1%, 10% and 50% of maximum intensity of the rocking curve is illustrated in Fig 6a–c. The FW1%M map highlights long-range effects of the 3C-SiC defect that cause significant peak broadening of the rocking curve in this region. These highly distorted regions degrade electronic properties of the material, forming traps and potentially non-radiative centres that lower carrier mobility, ultimately risking device performance .^14^ The FW10%M map reveals intermediate regions affected by the 3C-SiC defect, extending defect effects moderately farther from the defect compared to the range indicated by the FWHM. This range indicates a gradual dissipation of defect effects that impact material quality. The defective zones in the crystal form a small fraction of the whole; an enhanced contribution of defect-induced diffuse scattering at lower intensity levels of the rocking curve is more sensitive than, for example, FWHM, which is dominated by the quality of the bulk crystal. The FWHM reveals regions where the defect effects are minimal but detectable, representing a diffuse impact over a narrower range. A narrow FWxM is indicative of fewer structural defects, while a broader one suggests the presence of more defects.^16,18^ Therefore, the FWxM maps provide insight into the spatial extent and varying severity of the 3C-SiC defect’s impact on the 4H-SiC material quality. The maps highlight how the 3C-SiC defect not only disrupts the crystal lattice in its immediate vicinity, but also introduces a gradient of strain that impacts material quality at different level. Hence for defect analysis, we propose full width at 1% and 10% of maximum intensity over traditional FWHM measurements, as these approaches are more sensitive to crystal defects that causes broadening in the tails of the rocking curve.

#### White Beam Imaging

**Fig. 7:**
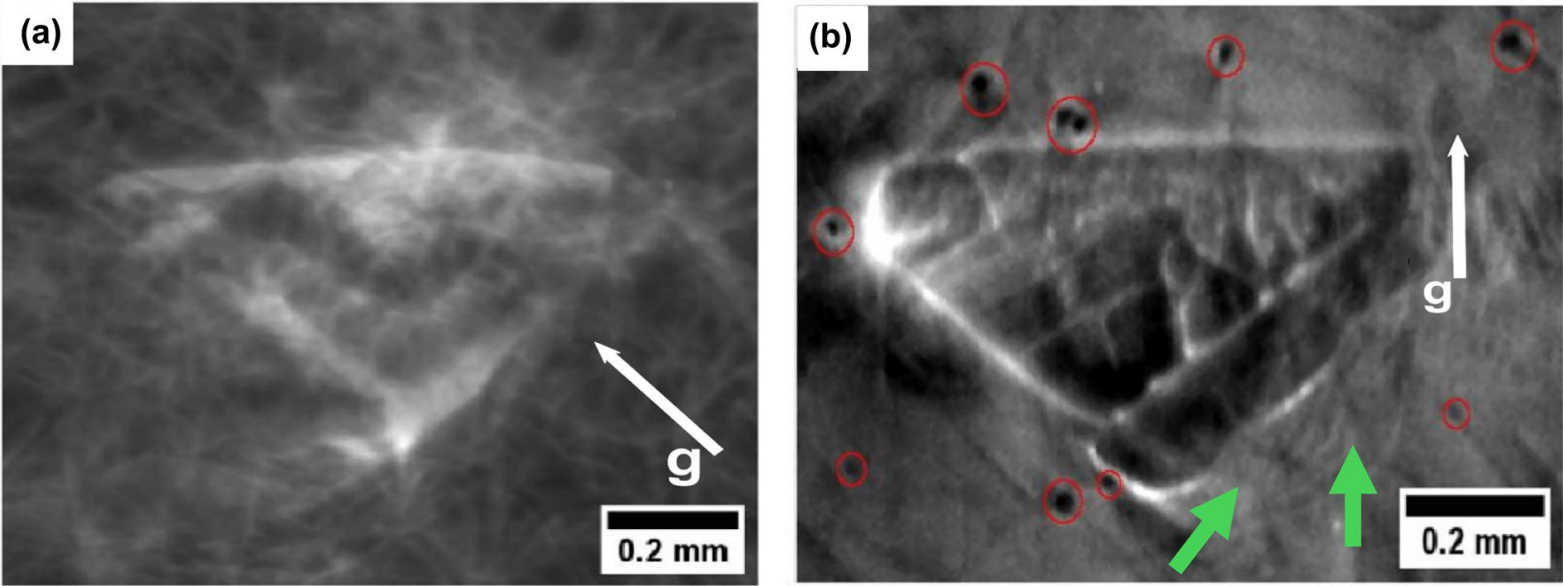
X-ray diffraction imaging showcasing the same 3C-SiC polytype in Fig. 5: white beam images (a) from $\left(1 1 \overline{2} 0\right)$ x4, (b) from $\left(3 \overline{3} 0 16\right)$ x4. Red circles indicate TSDs, and white arrows are g vectors.

Figure 7a and b present SWB-XRDI from the $\left(1 1 \overline{2} 0\right)$ and $\left(3 \overline{3} 0 16\right)$ crystal planes, respectively, showing the same 3C-SiC polytype highlighted in the red rectangles in Fig. 5a and b. Based on invisibility criteria when $\overrightarrow{\text{g}}.\overrightarrow{\text{b}}$ = 0 ($\overrightarrow{\text{g}}$ is the diffraction vector, represented by white arrows in Fig. 7a and b, and $\overrightarrow{\text{b}}$ is the Burger’s vector), the corresponding defect is invisible .^18^ In Fig. 7a, the white lines are threading edge dislocations (TEDs) and basal plane dislocations (BPDs). The small black dots encircled in red in Fig. 7b represent threading screw dislocations (TSDs), while the curved lines surrounding the 3C-SiC, shown by green arrows, indicate BPDs.

### Correlation of Features

**Fig. 8:**
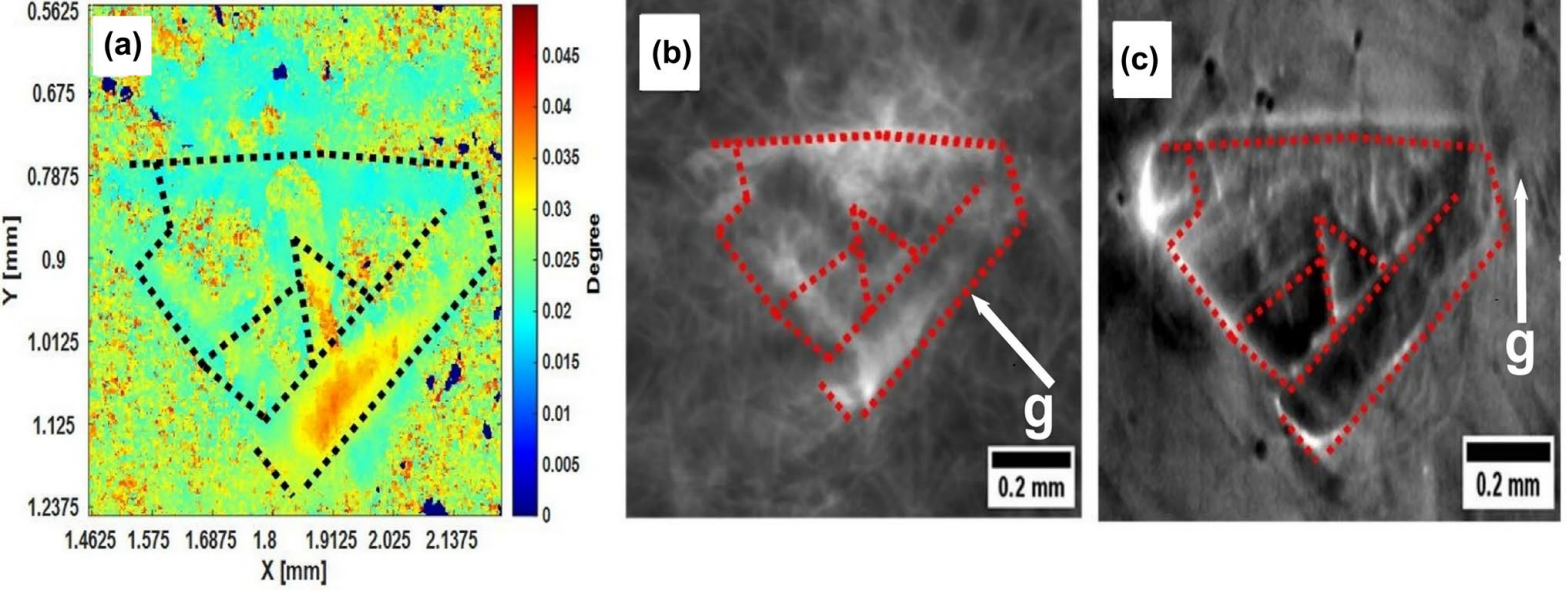
Identification of 3C-SiC polytype defect within 4H-SiC matrix using (a) CII for full width at 1% maximum (FW1%M), and synchrotron white beam imaging from (b) $\left(1 1 \overline{2} 0\right)$, and (c) $\left(3 \overline{3} 0 16\right)$. Black and red dotted lines highlight corresponding regions of the 3C-SiC polytype across images.

Figure 8 illustrates a comparative analysis of the 3C-SiC polytype within a 4H-SiC matrix and synchrotron white beam imaging. Figure 8a illustrates a detailed pixel by pixel peak broadening map of the full width at 1% maximum (FW1%M) that highlights subtle variations associated with the 3C-SiC defect. The overlaid dotted lines serve as visual guides, pinpointing areas where the 3C-SiC polytype disrupts the uniform structure of the 4H-SiC matrix.

Figure 8b and c, captured by SWB-XRT, provide high-resolution confirmation of this defect, with manually drawn dotted lines marking corresponding regions identified in Fig. 8a. The correlation between Fig. 8a–c validates the robustness of the CII method to accurately detect and quantify polytype-specific structural variations. This cross-validation underscores the utility of the CII approach for quantitative characterization and its potential for precise defect mapping.

### Rocking Curve Characteristics of Defects

**Fig. 9:**
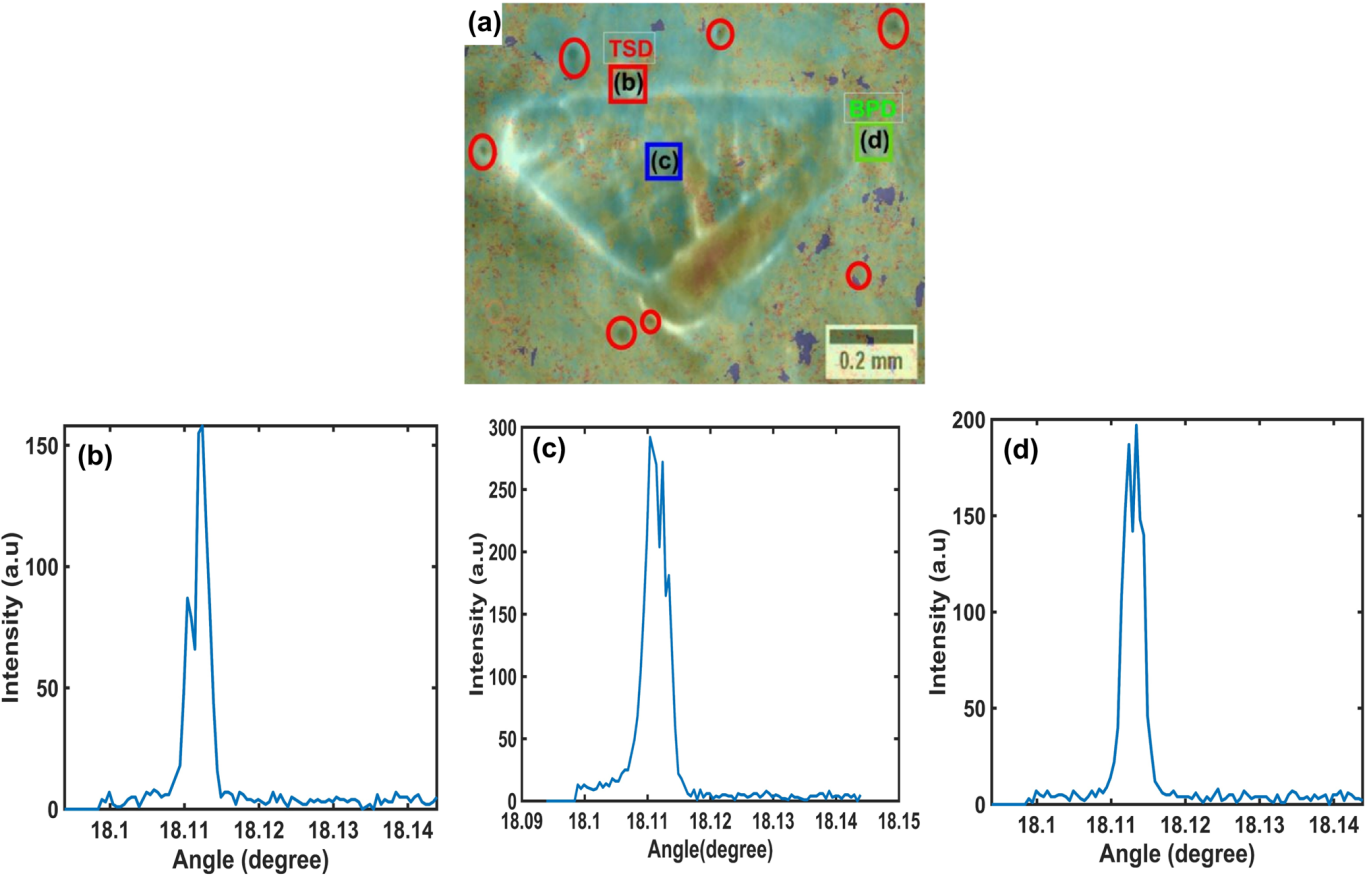
(a) Overlay of FW1%M map (Fig. 7a) and Fig. 8b, (b–d) RC-XRDI of the highlighted regions in (a) within the 3C-polytype defect.

Figure 9a displays an overlay of FW1%M from Fig. 8a and the SWB-XRDI from $\left(3 \overline{3} 0 16\right)$ presented in Fig. 7b. This overlay enables precise localization of TSDs, highlighted in red, and BPDs, highlighted in green. The labelled areas in Fig. 9a, denoted as (b), (c) and (d), correspond to specific regions around and within the 3C-SiC defect where rocking curve data have been extracted. These are presented in Fig. 9b–d for TSDs, the interior of the 3C-SiC defect and BPDs, respectively. A common feature among all these rocking curves is the presence of peak splitting and asymmetric peak shapes. Table III provides detailed measurements for FW1%M, FW10%M, and the peak positions detected by both the CII method and *findpeaks* across regions (b), (c) and (d). The negligible discrepancy between peak positions, differing by only 0.0005°, corresponding to one step size, demonstrates the accuracy and reliability of the CII method in peak detection, especially in scenarios dealing with split peaks. Additionally, the differences in rocking curve profiles across these regions reflect the varying interactions between the x-ray beams and different regions of the 3C-SiC defect, as well as their impact on the crystal lattice.

**Table III:** Values of FW1%M, FW10%M, and peak position by CII and *findpeaks* for different regions of 3C-SiC

Parameter	Region (b)	Region (c)	Region (d)
FW1%M by CII	0.0215°	0.0220°	0.0245°
FW10%M by CII	0.0205°	0.0205°	0.0220°
Peak position by CII	18.1119°	18.1109°	18.1129°
Peak position by *findpeaks*	18.1124°	18.1104°	18.1134°
Absolute difference of Peak position by CII and *findpeaks*	0.0005°	0.0005°	0.0005°

By analysing these rocking curves, we can infer the spatial distribution and severity of lattice imperfections. This detailed understanding is essential for characterizing defect-induced distortions, which impact different materials' properties

## Conclusion

The cumulative integrated intensity (CII) method offers a non-curve-fitting approach to analysing rocking curves, effectively handling non-Gaussian characteristics such as split and multiple peaks. This novel method offers a significant advantage by treating multiple and split peaks as a single peak, eliminating the need for complex algorithms to calculate the coefficient of determination and simplifying the analysis process. For detailed defect analysis, full width at 1% or 10% of maximum intensity are recommended over FWHM, since FWHM is dominated by bulk material, and local defects are likely to give diffuse scatter in the rocking curve tails. Correctly subtracted backgrounds yield CII tails that exhibit plateau behaviour, while over- or under-estimations distort the results. Furthermore, rocking curves extracted from various regions within the 3C-SiC polytype exhibit split peaks, indicative of underlying defect characteristics that merit further investigation. Overall, the implementation of this method has a high potential to enhance defect characterization and provides crucial insights for optimizing material performance in advanced applications.
